# Oral administration of VDAC1-derived small molecule peptides increases circulating testosterone levels in male rats

**DOI:** 10.3389/fendo.2022.1003017

**Published:** 2023-01-04

**Authors:** Daniel B. Martinez–Arguelles, Jennifer W. Nedow, Hovhannes J. Gukasyan, Vassilios Papadopoulos

**Affiliations:** ^1^ Department of Medicine, Research Institute of the McGill University Health Centre, McGill University, Montreal, QC, Canada; ^2^ Department of Pharmacology and Pharmaceutical Sciences, School of Pharmacy, University of Southern California, Los Angeles, CA, United States

**Keywords:** testosterone, Leydig cell, hypogonadism, aging, small molecule, therapeutics

## Abstract

Cholesterol is the precursor of all steroid hormones, and the entry of cholesterol into the mitochondria is the rate-limiting step of steroidogenesis. Voltage-dependent anion channel (VDAC1) is an outer mitochondrial protein part of a multiprotein complex that imports cholesterol. We previously reported that intratesticular administration of a 25 amino acid peptide blocking the interaction between 14-3-3ϵ with VDAC1 increased circulating levels of testosterone. This fusion peptide was composed of a HIV-1 transactivator of transcription (TAT) protein transduction domain cell-penetrating peptide, a glycine linker, and amino acids 159-172 of VDAC1 (TV159-172). Here, we describe the development of a family of small molecules that increase circulating testosterone levels after an oral administration. We first characterized an animal model where TV159-172 was delivered subcutaneously. This subcutaneous model allowed us to study the interactions between TV159-172 and the hypothalamus-pituitary-gonadal axis (HPG) and identify the biologically active core of TV159-172. The core consisted of the tetrapeptide RVTQ, which we used as a platform to design synthetic peptide derivatives that can be administered orally. We developed a second animal model to test various derivatives of RVTQ and found 11 active compounds. Dose-response experiments identified 4 synthetic peptides that robustly increased androgen levels in a specific manner. We selected RdVTQ as the leading VDAC1-core derivative and profiled the response across the lifespan of Brown-Norway rats. In summary, we present the development of a new class of therapeutics that act within the HPG axis to increase testosterone levels specifically. This new class of small molecules self-regulates, preventing abuse.

## 1 Introduction

Testosterone, made by the Leydig cells of the testis, drives the establishment and function of the male reproductive system from gestation to adulthood (1, 2). During gestation, fetal Leydig cells arise from mesenchymal precursors and produce high levels of testosterone before acquiring luteinizing hormone (LH) receptors (2, 3). Testosterone production declines with the loss of the fetal Leydig cells, and then gradually increases to high levels with the development of adult Leydig cells from stem cells (2, 3). Testosterone levels in men physiologically decrease at a rate of 0.4 to 2% per year starting at age 30 (4–6)

The signal to produce testosterone initiates in the hypothalamus with the release of Gonadotropin-releasing hormone (GnRH) into the hypophyseal portal circulation (7). GnRH stimulates the gonadotropic cells in the anterior pituitary gland to release luteinizing hormone (LH) (8). GnRH and LH are pulsatile and follow a circadian rhythm with testosterone levels being higher during morning hours (9). Upon activation of the LH receptor in Leydig cells, second messengers initiate the movement of cholesterol into the mitochondria to start steroidogenesis (2). Testosterone is eventually released into the circulation, where androgen receptors in the hypothalamus and pituitary mediate the negative feedback loop modulating GnRH and LH release (10).

Hypogonadism is the decrease of testosterone levels that is often accompanied by erectile dysfunction, decreased muscle mass, gynecomastia, and osteoporosis, as well as physiological symptoms like fatigue, mental fogginess, and decreased libido (11, 12). Primary hypogonadism occurs when Leydig cell androgen production is insufficient. However, in most of the cases, hypogonadism is secondary, where GnRH or LH signaling is inadequate to maintain testosterone levels (4). There is also a subgroup of males with a mix of central (hypothalamic and/or pituitary) and gonadal deficiency known as late-onset hypogonadism (6, 11).

Testosterone replacement therapy (TRT) is currently the only approved therapy to treat hypogonadism and uses synthetic testosterone analogs to improve the symptoms of patients (13–15). Side effects of TRT include polycythemia (16, 17), gynecomastia (18), and infertility (19). Other side effects, like cross-contamination in patients using gels (20, 21) and rashes (20) in those using patches, are associated with the route of administration. There are also concerns about suppression of reproduction and abuse. Moreover, cardiovascular concerns were raised in retrospective (22, 23) and prospective (24, 25) studies. However, some studies showed no effects or even cardiovascular improvements (26–28) calling for caution and long-term cardiovascular safety studies (29, 30). All these concerns lead the FDA to issue a warning and guidelines that aim to reduce the abuse of TRT (31). Abuse of TRT is also a concern, since improper use of TRT may affect the HPG axis up to 2-3 years and, in some cases, permanently (32–34).

Parenteral administration is an accurate way of dosing TRT (35), but users frequently miss doses and have the least compliance (36). Unfortunately, the oral route, which is shown to have the best compliance, has a limited therapeutic option due to liver toxicity (37) although there is recent progress in this area (38). The use of human chorionic gonadotropin (hCG) (39–41), modulators of the estrogen receptor (ER) like clomiphene citrate (41–44), and aromatase inhibitors (41, 45, 46), which block the conversion of testosterone into estrogen, have been proposed but are not currently endorsed by the FDA. Novel oral therapies, alternatives to current TRT methods, clearly are desirable (47, 48). To this end, we focused on the mechanisms of steroid biosynthesis, the basis for testosterone production.

We previously reported the development of a fusion peptide that increases steroidogenesis by targeting the machinery that imports cholesterol into the mitochondria (49). Cholesterol is the precursor of all steroid hormones and the movement of cholesterol into the inner mitochondria membrane is the rate-limiting step in steroidogenesis (1). Cholesterol transport and translocation is aided by the voltage-dependent anion-selective channel protein 1 (VDAC1) that is part of a multiprotein complex called the transduceosome (50–52). VDAC1 is a 19-transmembrane domain β-barrel protein located in the outer mitochondria membrane and known to exchange ions and metabolites bidirectionally (53). The rate of mitochondrial cholesterol import is controlled by interactions between VDAC1, steroidogenic acute regulator protein STAR (54), and translocator protein (18 kDa) TSPO (55, 56). Among the proteins associated with the transduceosome, the 14-3-3ϵ protein adaptor also interacts with VDAC1, forming a scaffold that limits the availability of cholesterol for steroidogenesis, thus acting as a negative regulator of steroidogenesis (49). We previously identified that amino acids 159-172 (based on NCBI Reference Sequence [RefSeq] NP_112643.1), located between the cytosolic 10th and 11th transmembrane loops of VDAC1, are likely mediating the 14-3-3ϵ-VDAC1 interaction, controlling cholesterol import and androgen biosynthesis (49). We previously showed that TVS167, a fusion protein composed of the HIV-1 transactivator of transcription (TAT) protein transduction domain- cell-penetrating sequence, a glycine linker, and amino acids 159-172 from VDAC1, increased testosterone levels (49, 57). TVS167 stands for TAT-VDAC1-Ser 167, an amino acid part of the VDAC1 motif predicted to be recognized by 14-3-3ϵ (49). We reported that testes from Sprague-Dawley rats, treated *ex vivo* with TVS167 and stimulated with hCG, had significantly increased testosterone levels. This androgenic effect was also seen *in vivo* after a 24 hr intratesticular TVS167 infusion (49). More importantly, TVS167 was able to recover testosterone production in a chemically castrated rat model (treated with the GnRH antagonist cetrorelix) (49).

**Figure 1:**
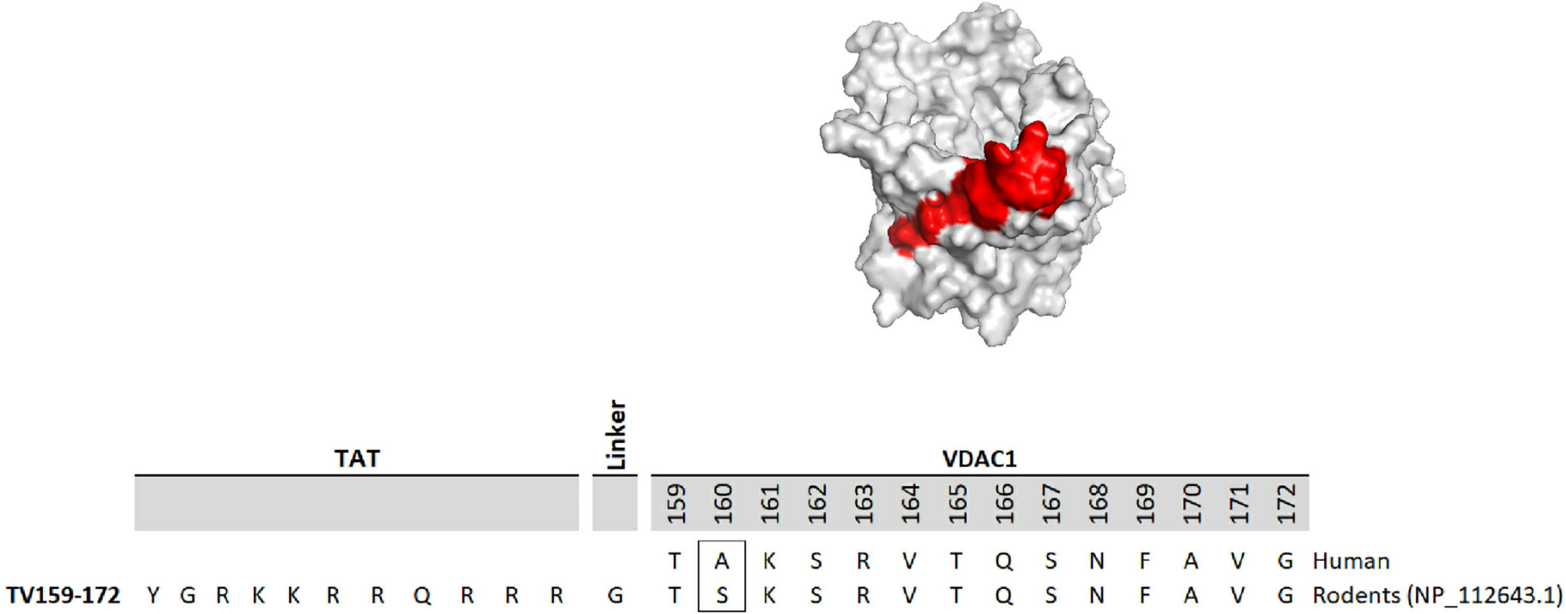
TV159-170 is a fusion peptide. The figure shows the human VDAC1 structure where the red depicts the VDAC1 amino acids contained in TV159-170, which are oriented towards the cytosol. The human VDAC1 protein sequence differs from the rodents only at amino acid 160.

The development of molecules that increase androgen levels without triggering the negative feedback loop regulating testosterone biosynthesis would be a major milestone. To precisely identify and emphasize the VDAC1 amino acids contained in the fusion protein based on VDAC1 RefSeq NP_112643.1, we changed the nomenclature of TVS167 to TV159-172 where the T denotes the TAT-cell penetrating sequence plus glycine linker (YGRKKRRQRRR+G) and V159-172 indicates the VDAC1-derived amino acids (TSKSRVTQSNFAVG). The VDAC1 sequence contained in TV159-172 is identical between rodents and humans except for amino acid 160 that in rodents is a serine and in humans is an alanine (**Figure 1**).

Here, we used a multi-step approach and various animal models to characterize the pharmacodynamics of TV159-172, identify the minimum bioactive sequence, and characterize a new family of oral peptides that promote increases in circulating testosterone levels. First, we identified the TV159-172 dose range associated with increases in circulating testosterone levels and established that LH signaling is needed for peptide function using a subcutaneous delivery animal model in the Sprague-Dawley rat. Next, we used sequential peptide deletions to identify RVTQ as the bioactive sequence of TV159-172 using a subcutaneous delivery model in the Brown-Norway rat. The deletion data also suggested that the TAT-cell penetrating tag could be excluded since the RVTQ core was within the molecular weight of a small molecule. These findings led us to explore the oral administration of the peptides as a novel route for peptide delivery. To increase the pool of candidate molecules, we identified the critical amino acids in the peptides using evolutionary biology and expanded the candidate pool by introducing modifications to the core peptides during synthesis. These candidate molecules were tested in an oral animal model in the Brown-Norway rat, which we devised to rapidly screen and optimize orally active peptides. We identified several bioactive oral molecules and ultimately selected a lead molecule with robust characteristics for further characterization across the age range of the Brown-Norway rat. This body of work resulted in the identification of a new class of small molecules that increase androgen production after oral administration while remaining under HPG axis control.

## 2 Material and methods

### 2.1 Reagents

Peptides were obtained from CanPeptide (Montreal, Quebec) with >95% purity. All peptides were dissolved in molecular grade, sterile double distilled water to obtain a 1 mM stock solution and stored at -20°C. Peptides used for oral screenings were prepared from 1 mM peptide stock and diluted in sterile tap water in a total volume of 1 ml. Peptides used for pharmacokinetic experiments were diluted at 10 mg/ml using sterile tap water.

Human chorionic gonadotropin (hCG, specific activity 6000 UI/ml) was obtained from. National Hormone & Peptide Program, Harbor-UCLA Medical Center, Torrance, CA. Adrenocorticotropic hormone fragment 1-24 human (ACTH) was purchased from Sigma-Aldrich (St Louis, MO) (Cat#A0298).

### 2.2 Osmotic pumps

Osmotic pumps were purchased from Alzet (Cupertino, CA) with the following infusion rates Alzet 2001, 1 μL/hr; Alzet 2006, 0.15 μL/hr; and Alzet 1007D, 0.5 μL/hr. The peptide concentration loaded into the pumps accounted for weight gain at the time of collection and was adjusted to deliver the doses indicated in each figure. The pump infusion rates varied by production lot and were adjusted accordingly. The electronic programmable pump SMP-200 was purchased from Alzet and programmed using the data communication device UCD-200 and the iPRECIO Management Software (Alzet, Cupertino, CA), as indicated by the manufacturer’s instructions. Pumps were loaded under sterile conditions 24 hr before surgical implantation and were kept at 37°C in PBS until their use.

### 2.3 Animal handling, blood collections, surgical procedures, and oral administration of peptides

Sprague-Dawley rats 50-55 days old were obtained from Charles River (Senneville, Quebec). Brown-Norway rats aged 22-28, 36-42, or 50-56 days-old were purchased from Charles River Laboratories (Senneville, Quebec) and aged in our own facility until needed. Some of the older (over 12 months old rats) were provided by the National Institute of Aging (NIH, Bethesda, MD, USA). Rats were kept on a 12L/12D day cycle with lights on at 7 AM and access to food and water *ad libitum*. Animals were handled according to protocols approved by the McGill University Animal Care and Use Committee, which included standard operation procedures for repetitive jugular collections.

Plasma samples were obtained *via* percutaneous jugular puncture and collected in EDTA KE/1.3 tubes (Sarstedt, Numbrecht, Germany, Cat# 5072511). Blood collection time points are indicated in each figure legend. Collection of blood from cardiac puncture was a terminal procedure. The animals were first anesthetized with isoflurane followed by CO_2_ and blood collection occurred soon after no reflexes were detected. Cardiac puncture samples were collected in uncoagulated tubes (serum). Both types of collections were centrifugated at 1,300 RCF for 10 min and stored in 2 ml Wheaton glass vials (Fisher Scientific, Hampton, NH, Cat# 03 337 21A) and kept at -20°C.

Intratesticular fluid was collected by placing the decapsulated left testes in 1 ml of PBS for 30 min at 4°C in a 50 ml Falcon tube. The tubes were then centrifuged at 2,200 RPM for 2 min. and the supernatant was collected and stored as noted above.

Sprague rats age ~55 day-old or Brown-Norway rats aged ~29 and ~60 day-old were implanted with osmotic infusion pumps in the interscapular region under general anesthesia with isoflurane following antisepsis using iodine. The surgical wound was closed with two to three staples that were removed a week after the surgery. Subcutaneous carprofen (Zoetis, Parsippany, NJ) was given before the surgical procedure and in the following two days for pain management. For experiments requiring pump changes, a surgical incision was made 2 cm below the original wound from which the consumed pump was removed and the new one implanted. Staples and pain managements were carried out as described above.

For all oral experiments, administration *via* gavage began between 8:30-8:50 am with a 3-minute delay between each rat. Animals used for pharmacokinetic studies were fasted for 4 hrs before starting gavage at 1 pm with a 3-minute delay between each rat.

### 2.4 Plasma steroid measurements

**Figure 2:**
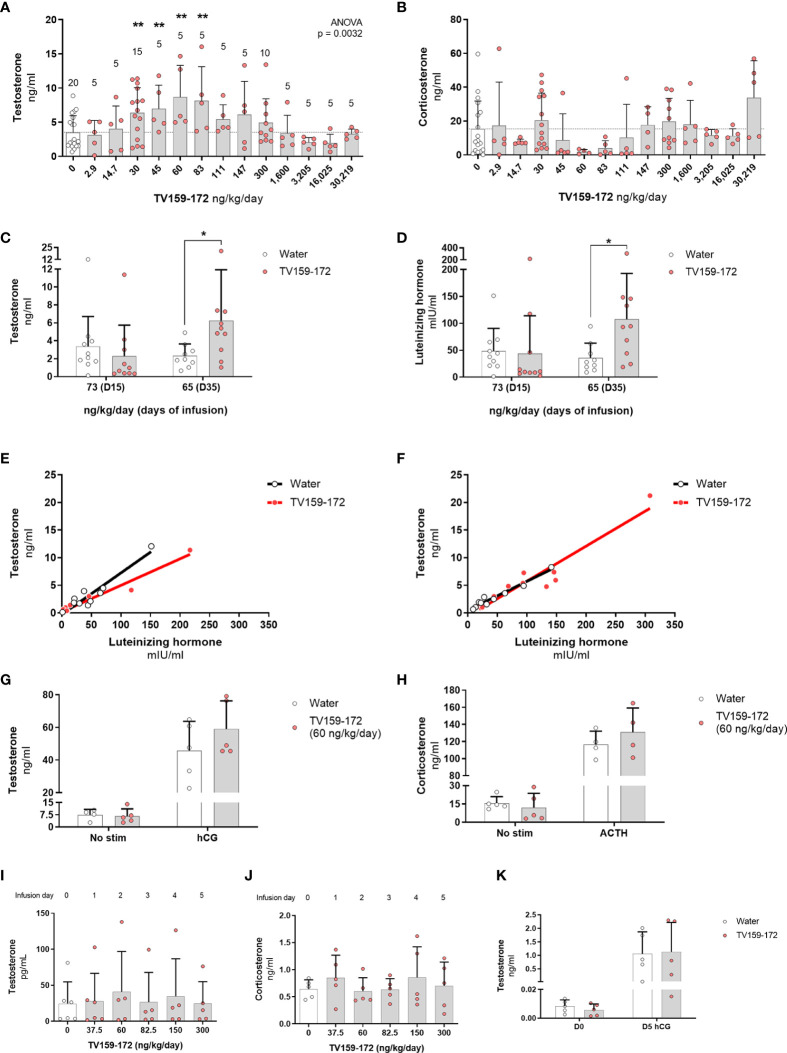
**(A, B)** Subcutaneous infusion of TV159-172 increases testosterone levels. Sprague-Dawley rats were implanted with osmotic pumps delivering various concentrations of TV159-172 for 1 week with graphs showing the results of three pooled rounds. Testosterone **(A)** and corticosterone **(B)** levels were measured in serum at the end of the treatment. Data shown represent means ± standard deviation. The N is depicted on top of each bar. p*< 0.05, p**<0.01. **(C–F)** Chronic infusion of TV159-172. Sprague-Dawley rats were implanted with osmotic pumps and plasma samples were collected 15 and 35 days after implantation. Plasma testosterone **(C)** and luteinizing hormone **(D)** levels are shown. **(E**, **F)** Correlation between testosterone and luteinizing hormone levels in samples collected at infusion day 15 and 35. Data shown represents means ± standard deviation (N=10); p* < 0.05. **(G, H)** Stimulation with supraphysiological levels of hCG or ACTH. Sprague-Dawley rats were implanted with subcutaneous osmotic pumps delivering TV159-172 for 1 week. Serum testosterone **(G)** and corticosterone **(H)** levels from rats not stimulated or stimulated with hCG or ACTH for 90 min are shown. Data shown represent means ± standard deviation (N=5). **(I–K)** Effects of TV159-172 in hypophysectomized rats. Plasma testosterone **(I)** and corticosterone **(J)** levels in Sprague-Dawley rats implanted subcutaneously with programmable pumps delivering increasing concentrations of TV159-172 every 24 hrs. **(K)** Serum testosterone levels in hypophysectomized rats infused with TV159-172 for one week and stimulated with hCG for 90 min. Data shown represent means ± standard deviation (N=5).

Testosterone levels were measured using Cayman (Ann Arbor, MI) EIA kit Cat# 582701 (RRID : AB_2895148); dihydrotestosterone levels were measured using CUSAbio (College Park, MD) kit Cat# CSB-E07879r; luteinizing hormone levels were measured using CUSAbio kit Cat# CSB-E12654r; estradiol levels were measured using CUSAbio kit Cat# CSB-E05110r; corticosterone levels were measured using CUSAbio kit Cat# CSB-E07014r for **Figure 2** data and Cayman EIA kit Cat# 501320 (RRID : AB_2868564) for the remainder experiments; and aldosterone levels were measured using Cayman EIA Cat# 10004377 (RRID : AB_2895148). All samples were measured in duplicate according to each of the manufacturer’s instructions. Serum, plasma, or intratesticular fluid samples were diluted until controls fitted the middle of the standard curve used. The number of animals used for each experiment is noted in the corresponding figure legend.

### 2.5 Hypophysectomized rats

Hypophysectomized Sprague-Dawley rats were purchased from Charles River and were given water supplemented with 5% glucose ad libitum. Animals had the pituitary glands surgically removed by Charles River staff one week before their arrival at our animal facility. Basal testosterone levels were taken *via* jugular puncture 3 days after their arrival and prior surgical implantation of SMP-200 or 2001 Alzet pumps.

In experiments involving SMP-200 pumps, the animals were allowed to recover for 68 hrs post-surgical implantation before the start of daily blood collections *via* jugular puncture. SMP-200 pumps were loaded with 380 ng/ml of TV159-172 and were programmed to delivery 0.5 µL/hr for 68hr then progressively increased to 1, then 1.6, 2.2, 4, and finally 8 µL/hr every 24hrs. In experiments involving Alzet 2001 pumps, animals were infused for seven days at the levels noted. Animals were stimulated with 150 UI hCG intraperitoneally in the last infusion day and samples were collected 2 hrs after *via* cardiac puncture.

### 2.6 hCG and ACTH stimulation

Sprague-Dawley rats were subcutaneously implanted with Alzet 1007D pumps delivering water or 60 ng/kg/day of TV159-172 (**Figures 2G, H**). After 5 days, some rats were stimulated with either 150 UI hCG intraperitoneally or 0.5 mg ACTH/kg. This resulted in 6 experimental groups (N = 5), which were water, water + hCG, water + ACTH, TV159-172, TV159-172 + hCG, and TV159-172 + ACTH. Final blood collections were taken 90 min post- hCG and ACTH stimulation *via* cardiac puncture.

### 2.7 Peptide stability in plasma, oral pharmacokinetics, and quantification

For peptide stability experiments, plasma from Brown-Norway rats aged 90-140 days was collected and stored at -20°C until further use. The plasma was thawed, spun at 12,000 RPM for 10 min, and kept at 4°C. 4 μM of the various peptides were incubated with 100 μL of plasma that was warmed at 37°C for 10 min before use. After 1, 15, 30, 60, 90 or 120 minutes, the plasma/peptide samples were crashed with 300 µL of 100% methanol, spun at 14,000 RPM for 10 min, and the supernatant collected. The methanol in the supernatant was evaporated and the samples were re-extracted. The final pellet was solubilized in 5 μL methanol followed by the addition of 100 μL of sterile double distilled water. The extracted samples were transferred to plastic tubes and placed in the autoloader of the mass spectrometer.

For oral pharmacokinetic experiments, jugular blood samples were collected following a single oral dose of 10 mg peptide in 1 ml of vehicle after 5, 15, 30, 45, 60, 90, and 120 minutes. Protein inhibitor cocktail (ROCHE, cOmplete protease inhibitors, Cat# 11697498001), which was added into the EDTA collection tubes, was used for KVSQ and RVTQ peptides. Plasma was immediately extracted, and 100 μL of plasma were crashed with 300 µL of 100% methanol, spun at 14,000 RPM for 10 min, and the supernatant harvested. Re-extraction of the precipitant and solubilization of the pellet were as described above.

The analytical instrumentation consisted of a Thermo-Scientific ISQ Quantiva Triple Quadrupole Mass Spectrometer (QQQ), incorporating a heated electrospray ionization (HESI) source, and a Thermo-Scientific UltiMate™ 3000 UHPLC system (including, UltiMate™ 3000 RS autosampler). Analytical conditions were developed using a reference standard in a solution containing 5 µM of the corresponding peptide. The chromatography was resolved by isocratic elution of a binary solvent system incorporating (A) 0.1% Formic Acid (aq) and (B) ACN + 0.1% FA and using an Agilent Eclipse Plus™ C18 analytical column (100mm X 2.1mm ID, 1.8 µm particle). A 5µL injection volume and solvent flow rate of 150 µL/min were used. The MS/MS acquisition time was 7 min and total run time was 8.3 min/injection. The triple Quadrupole MS/MS instrumentation conditions were as follows: the HESI source voltage was 3200 V, the sheath gas 30 L/min, auxilirary gas 20 L/min, sweep gas 2.0 L/min, the ion transfer temperature was 350°C, the vaporizer temperature was 350°C, the CID gas was 1.5 (mTorr) and the dwell time was 100 msec. The Q1 Resolution (FWHM) was 0.4 (unitless) and the Q3 resolution (FWHM) was 0.7 (unitless). The instrument ran in a selected reaction monitoring (SRM), positive ion detection mode at various collision energies (CE) for the following mass transitions:

Ac-RITQdS-CONH_2_ (*m/z* 645→*m/z* 295, *m/z* 396) (N-terminal acetylation and C-terminal amidation).

RdVTQ (*m/z* 503→*m/z* 211).

Ac-RdITQ-CONH_2_ (*m/z* 558→*m/z* 391, *m/z* 267)

RVTQ (*m/z* 503→*m/z* 211; *m/z* 503→*m/z* 357 and *m/z* 503→*m/z* 256)

KVSQ (*m/z* 461→*m/z* 129) and (*m/z* 461→*m/z* 234)

### 2.8 Peptide modeling

VDAC1 from the Protein Data Bank accession number 5JDP was used for modeling in PyMOL (Schrodinger, Cambridge, MA). Physicochemical properties of peptide were calculated used ADMET Predictor v. 10.3 (SimulationsPlus, Lancaster, CA).

### 2.9 Statistical analysis

GraphPad Prism 7.04 (GraphPad Software, La Jolla, CA) and Excel 2006 (Microsoft Corporation, Redmond, WA) were used to generate graphs, heatmap, curve fitting, and statistical analysis. ANOVA or a one-tail t-test was used to determine significant changes. The number of animals used per experiment is noted in each of the corresponding figure legends. Data are shown as mean ± standard deviation unless otherwise specified in the figure legend.

## 3 Results

### 3.1 Subcutaneous delivery of TV159-172 increases testosterone levels in young adult Sprague-Dawley rats

To elucidate the effects of a continuous infusion of TV159-172 on intratesticular and circulating steroid levels, we surgically implanted subcutaneous osmotic pumps that delivered increasing doses of TV159-172. Serum steroid levels measured after 7 days of treatment showed increases, although not significant, in intratesticular testosterone levels in animals receiving 30 ng/kg/day TV159-172 (**Figure S1A**). The stimulatory effect of TV159-172 was suspected to be around the 30 ng/kg/day dose, because similar increases were observed in circulating levels of testosterone (**Figure S1B**) and dihydrotestosterone (**Figure S1C**), a potent testosterone metabolite. LH levels mirrored those of intratesticular and serum testosterone at the 30 ng/kg/day/dose (**Figure S1D**). Serum corticosterone levels were also increased, showing a non-significant effect at the 30 and 300 ng/kg/day doses (**Figure S1E**). Serum estradiol and aldosterone levels were comparable to controls (**Figures S1F, G**).

We conducted a detailed dose-response study, in three rounds, to further characterize the increases in circulating serum testosterone and corticosterone levels observed with the 30 ng/kg/day dose of TV159-172. As before, osmotic pumps were implanted subcutaneously, and testosterone and corticosterone levels were measured 7 days later. The pooled data showed a dose-dependent increase in testosterone levels with significant changes observed at 30-83 ng/kg/day (**Figure 2A**). No significant changes were observed in circulating corticosterone levels (**Figure 2B**).

### 3.2 Prolonged subcutaneous delivery of TV159-172 has mixed effects on testosterone levels

To characterize the effect of a prolonged infusion of TV159-172 on plasma testosterone and LH levels, we placed subcutaneous osmotic pumps in Sprague-Dawley rats delivering TV159-172 for up to 42 days.

Plasma testosterone levels collected after 15 days of infusion showed no significant changes. However, we noted that most of the treated rats showed a pattern of testosterone suppression with most samples showing very low levels of testosterone. Significant changes in testosterone were observed 35-days post-pump implantation corresponding to a dose of 65 ng/kg/day (**Figure 2C**). Plasma LH levels mirrored those of testosterone in control and treated rats at both time points with significant changes found at infusion day 35 (**Figure 2D**). The levels of testosterone and LH in treated animals showed a linear correlation in samples taken at infusion day 15 and 35 (**Figures 2E, F**, respectively). The high correlation between LH and testosterone levels suggests that TV159-172 likely acts within the HPG.

### 3.3 LH is needed for the steroidogenic effects of TV159-172

To further explore the interaction between TV159-172 and the HPG, we characterized steroid levels using a maximal LH stimulation or by ablating LH signaling.

We studied the effect that a 7-day TV159-172 infusion has on serum steroid production following suprastimulation with LH or ACTH. Sprague-Dawley rats were implanted with subcutaneous pumps delivering on average 59.2 ng/kg/day at the time of measurement. The results show that stimulation with hCG or ACTH has no significant effect on the serum levels of testosterone or corticosterone in animals treated with and without TV159-172 (**Figures 2G, H**). A discrete increase in testosterone levels, although not significant, was observed in treated rats post LH stimulation.

To gain insights into whether TV159-172 increased serum testosterone levels independent of LH, we implanted hypophysectomized Sprague-Dawley rats with a subcutaneous, programmable electronic pump delivering increasing concentrations of TV159-172 every 24 hr. The rats were sampled prior to pump implantation, representing dose 0. No increases in serum testosterone or corticosterone levels were observed at the doses tested (**Figures 2I, H**).

We stimulated an additional batch of hypophysectomized rats with 150 UI hCG to confirm that the Leydig cells still produced testosterone by the end of our previous experiment. We placed infusion pumps delivering 60 ng/kg/day at the time of collection. The results show that the hypophysectomized rats retained, although at decreased levels, their steroidogenic capacity with no significant changes observed in serum testosterone levels (**Figure 2K**).

Taken together, these data suggest that LH is needed to mediate the effects of TV159-172 and that TV159-172 does not increase the testosterone biosynthesis capacity, but rather, boosts LH signaling likely by increasing cholesterol import into mitochondria.

### 3.4 Derivatives of TV159-172 increase testosterone and corticosterone levels in Sprague-Dawley rats

**Figure 3:**
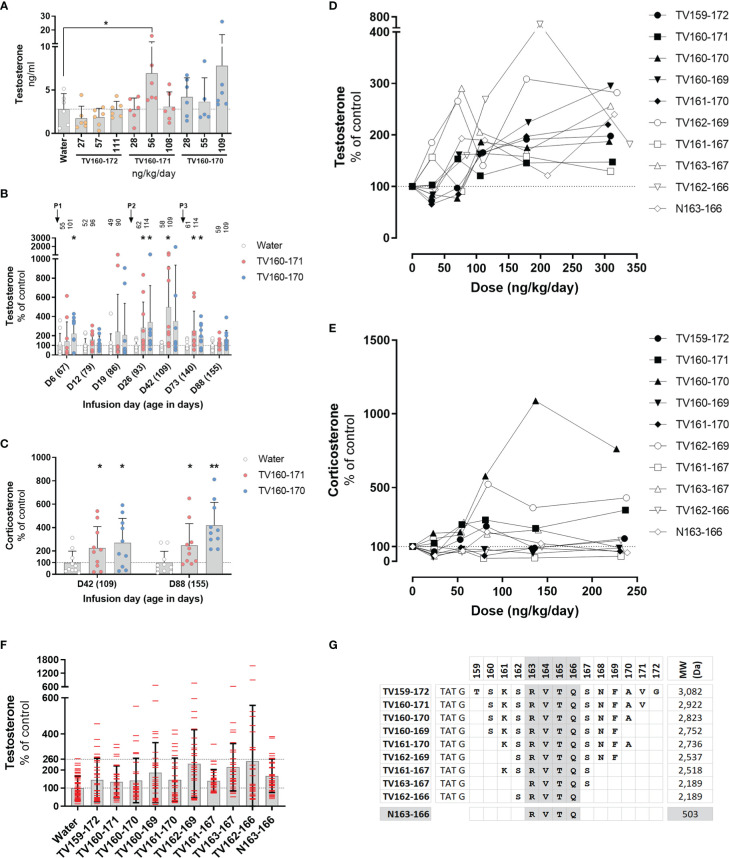
**(A–C)** TV160-171 and TV160-170 increase steroid levels. **(A)** Plasma testosterone levels in Sprague-Dawley treated with various concentrations of TV160-172, TV160-171, and TV160-170 for 1 week. **(B)** Plasma testosterone levels in Sprague-Dawley rats treated chronically with TV160-171 and TV160-170. The arrows depict the subcutaneous implantation of the infusion pumps. Peptide levels (ng/kg/day) delivered at the time of sampling are depicted on top of each bar. **(C)** Corticosterone levels in plasma samples from rats treated with TV160-171 and TV160-170 collected at infusion day 42 and 88. Data shown represents means ± standard deviation (N = 10); p*< 0.05, p**<0.01. **(D–F)** Deletions of TV159-172 retain androgenic activity. **(D)** Testosterone plasma levels in Brown-Norway rats implanted with subcutaneous pumps delivering increasing concentrations of indicated TV159-172 peptides. **(F)** Pooled testosterone levels of all water-treated rats and each peptide, independent of the dose. Data shown represents means ± standard deviation (N=6); p* < 0.05, p**<0.01. **(G)** Peptides were composed of TAT sequence, glycine linker, and the VDAC1-derived sequence shown in bold.

To study whether shorter peptides derived from TV159-172 have androgenic effects, we measured plasma testosterone levels in Sprague-Dawley rats receiving TV160-172, TV160-171, and TV160-170 subcutaneously for 1 week. The results show that TV160-171 significantly increased plasma testosterone levels at 56 ng/kg/day and TV160-170 at 109 ng/kg/day (**Figure 3A**).

We characterized the effect of an 88-day infusion of TV160-171 and TV160-170. Subcutaneous pumps were surgically placed at the start of the experiment when rats were 67 days old and changed at infusion days 24 and 66. Plasma testosterone levels were significantly increased at infusion days 6, 26, 42, and 73 in one or both peptides tested (**Figure 3B**). Some of the rats exhibited testosterone levels upwards of 10 times those of controls. Significant increases in testosterone were lost by continuous infusion day 88 in both peptides tested.

We sampled plasma corticosterone levels at infusion day 42, where testosterone levels were the highest, and at the end of the experiment, infusion day 88, where no significant changes in testosterone were observed. **Figure 3C** shows that corticosterone levels were significantly increased at both time points.

### 3.5 Shorter derivatives of TV159-172 are bioactive in inducing steroid formation

To continue the development of TV159-172, we switched to Brown-Norway rats because, compared to Sprague-Dawley rats, they have a less pronounce growth curve that plateaus around 350 gr, making continuous subcutaneous dosing easier to control. We implanted various groups of Brown-Norway rats with subcutaneous pumps delivering increasing doses of TV-peptides for 42 days. We conducted progressive single amino acid deletions from the N- and C- terminus until arriving at our shortest, TV163-167 and TV162-166, peptides that contained 5 amino acids from the original 14 amino acid VDAC1-derived sequence contained in TV159-172. Testosterone measurements in blood plasma collected 1 week after implantation showed significant increases in testosterone levels in TV156-172, TV160-169, TV161-170, TV162-169, TV161-167, TV162-166 (**Figure 3D**).

Plasma corticosterone levels collected around infusion day 40 were significantly increased by exposure to TV160-171, TV160-170, and TV162-169, but significantly decreased in response to TV161-167 treatment (**Figure 3E**).

Pooling of all testosterone control data and from all rats treated with TV-peptides, independent of the dose, showed an increased dispersion in all the TV-peptides (**Figure 3F**). The pooled results show that while the highest control reached 2.6 times those of the control average, rats receiving TV-peptides often had levels higher than 2.6-fold and, in some cases, upwards of 5 times those of control averages.

### 3.6 The tetrapeptide N163-166 increases testosterone levels

The deletion experiments showed that the shorter TV163-167 and TV162-166 peptides were steroidogenically active. **Figure 3G** shows that TV163-167 and TV162-166 overlap at amino acids 163-166 whose RVTQ sequence corresponds to the weight of a small molecule of 503 Daltons. To test whether the tetrapeptide was steroidogenically active, we subcutaneously implanted Brown-Norway rats with increasing concentrations of N163-166, where the N denotes the “naked” and TAT-less feature of this molecule. Testosterone and corticosterone levels were measured 7 and 40 days after implantation, respectively. The results obtained show that N163-166 significantly increased testosterone levels at 309 ng/kg/day (**Figure 3D**). Corticosterone levels were not affected by the treatment (**Figure 3E**). **Figure 3G** shows a summary of the deletion experiments that identified RVTQ as the bioactive core sequence (**Figure 3G**).

### 3.7 Subcutaneous infusion of RVTQ and its evolutionary-related sequences increase circulating testosterone levels

**Figure 4:**
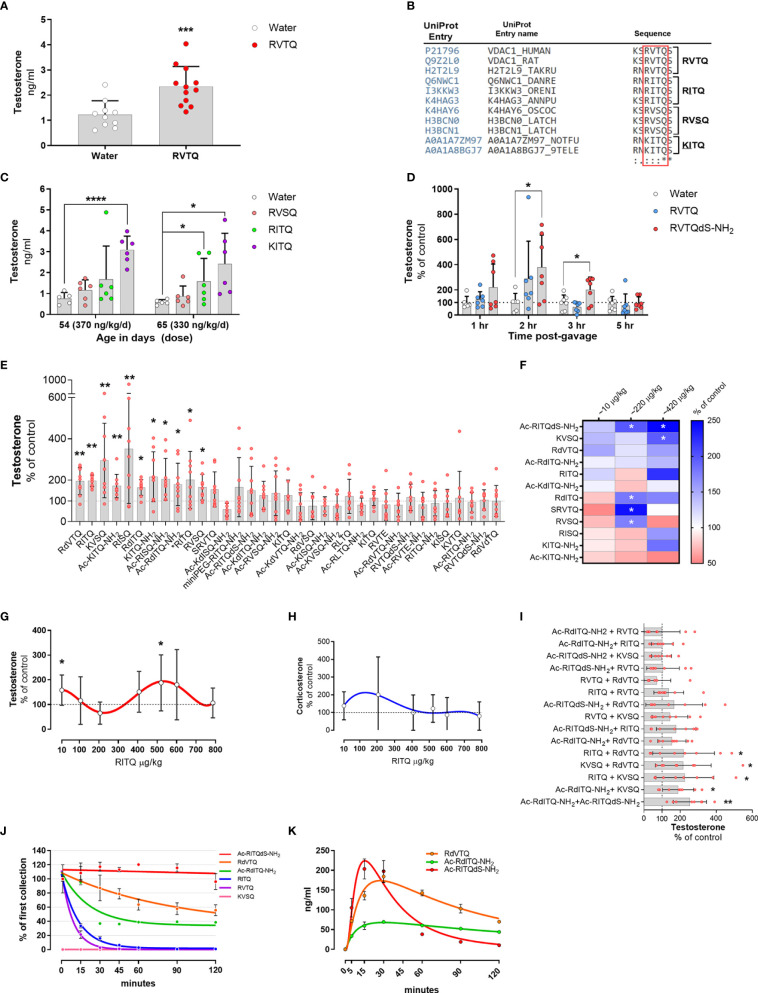
**(A–E)** Subcutaneous infusion of VDAC1-derived peptides increase plasma testosterone levels. **(A)** Testosterone levels in 54-day-old Brown-Norway rats implanted with osmotic pump delivering 377 ng/kg/d of N163-166 (RVTQ); N = 12; results shown as mean ± SD; ***p < 0.001. **(B)** Protein sequence alignment identifying the amino acid variations (underlined) of RVTQ in other species. **(C)** Testosterone levels in 54- and 65-day-old Brown-Norway rats infused with RVSQ, RITQ, or KITQ. N = 6; results shown as mean ± SD; ****p < 0.0001, *p<0.05. **(D)** Pool data showing time course of testosterone levels obtained in two rounds, 1 & 3 hrs and 2 & 5 hrs, post-gavage with RVTQ or RVTQdS-CONH_2_. Age of Brown-Norway rats during experiment was 60-123 days-old; N = 7; results shown as mean ± SD; *p<0.05; T denotes the order with which the peptides were tested. € I) Normalized testosterone levels of all peptides in the order tested. Age of Brown-Norway rats during experiment was 60-136 days-old; N = 8; results shown as mean ± SD; *p<0.05; **p<0.01; T denotes the order with which the peptides were tested. **(F)** Identification of candidate peptides with robust profiles. Heat map depicting the mean percent plasma testosterone levels with respect to control levels in rats 2 hrs after treatment with the various peptides and concentrations. Rats treated with ~10 and 220 µg peptide/kg had N = 7 and N=6 for ~420 µg peptide/kg. Age of Brown-Norway rats during experiment was 82 - 131 days-old; N = 8; *p<0.05. **(G, H)** Dose-response shows biphasic response in some peptides. **(G)** Testosterone and **(H)** corticosterone levels of Brown-Norway rats 2 hrs after gavage with the various concentrations of RITQ. Age of Brown-Norway rats during experiment was 88-138 days-old; N = 8; results shown as mean ± SD; *p<0.05. **(I)** Combination of 2-peptides given at very low doses increase plasma testosterone levels after oral administration. Testosterone levels in Brown-Norway rats aged 91-134 days-old treated with 2-pepdie combinations at ~10 µg peptide/kg. Normalized testosterone levels of all peptides in the order tested. N = 8; results shown as mean ± SD; *p<0.05; **p<0.01. T denotes the order with which the peptides were tested. **(J)** Modifications to the peptide cores increase plasma stability. % of peptide levels with respect to the first time point (1 min). N = 3; results shown as mean ± SEM. **(K)** Pharmacokinetics after single oral dose of selected peptides. Circulating levels after single oral dose of the various peptides. Age of Brown-Norway rats during experiment was 71 - 110 days-old; N = 4 in two rounds; Results shown as mean ± SEM.

We tested the permissiveness of the core sequence to modifications with the aim of expanding the pool of active sequences. We first corroborated the androgenic effects of the RVTQ (N163-166) core. Brown-Norway rats were implanted with subcutaneous infusion pumps at ~29 days old. Blood samples collected when the rats were ~60 days old, and those receiving a peptide concentration of 377 ng/kg/day showed a significant increase in testosterone levels (**Figure 4A**).

To identify potential amino acids that would accept modifications, we used the UniProt database and Clustal Omega to search for sequence variants of the N163-166 core found in nature (58). We identified RVSQ, RITQ, and KITQ (underline denotes the amino acid changes) as variations of the core sequence found mainly in fish and worms (**Figure 4B**). The sequence alignment also showed that the N-terminus amino acid flanking the N163-166 core was occupied by a serine or asparagine while the amino acid flanking the C-terminus was a serine, which was conserved in all the analyzed sequences. Moreover, all the amino acid changes identified were substituted for structurally similar amino acids.

To test whether RVSQ, RITQ, or KITQ increased testosterone levels, we implanted ~27-day old Brown-Norway rats with subcutaneous infusion pumps delivering the three evolutionary core variants. **Figure 4C** shows that samples collected at age 54 days, where the dose of KITQ reached ~370 ng/kg/day, had a significant increase in circulating testosterone levels. Samples collected 11 days after, at age 65 days when the concentration delivered was ~330 ng/kg/day, also showed a significant increase in RITQ. These results show that the core RVTQ is permissible to modifications and may allow for modulation of the target site.

### 3.8 Oral delivery of small molecule peptides increases testosterone levels

To ascertain whether RVTQ or its modified derivatives can be delivered orally, we developed an animal model where we could screen various modifications efficiently and identify those that were orally active. We tested six modifications to the RVTQ core in Brown-Norway rats aged 60 to 123 days old, which were gavaged in the morning, followed by jugular blood sample collection 3 hrs later. We used RVTQ as control and tested modifications that included the addition of a flanking D-serine, amide cap at the carboxy terminus and the addition of miniPEG or an acetyl cap at the N-terminus. The modifications were tested at random with doses ranging from 160 to 1,200 µg/kg (**Figures S2A–F**). **Figure S2C** shows that, RVTQdS-CONH_2_, composed of the core RVTQ sequence plus D-serine and an amide cap at the carboxy terminus, administered at ~580 µg/kg, was the combination of dose and modification that significantly increased testosterone levels. While RVTQdS-CONH_2_ increased testosterone levels, the naked RVTQ sequence did not show a significant effect at the 3 hrs sampling time.

We then ascertained the best sampling time to identify significant increases in testosterone after oral administration. We treated rats in two rounds with ~580 µg/kg of RVTQ or RVTQdS-CONH_2_ and collected blood samples at 1 and 3 hrs or 2 and 5 hrs (**Figure 4D**). The pooled data showed a time-dependent increase in testosterone levels that were significant at 2 and 3 hrs post-treatment. The data showed treatment with RVTQdS-CONH_2_ induced the highest levels of testosterone after 2 hrs. Based on these data, we adjusted the dose and collection time points of further screenings to ~550 µg/kg and 2 hrs, respectively.

### 3.9 Oral treatment with modified derivatives of RVTQ and evolutionary-related core sequences increase circulating testosterone levels

We hypothesized that the various changes to the core would yield a pool of molecules with diverse pharmacokinetic profiles with some favoring the oral route. Since the peptide acts within the HPG axis and is subject to negative feedback regulation, we also hypothesized that a derivative of the native RVTQ sequence would increase the chances of identifying a compound that stimulates testosterone production without triggering the HPG feedback regulation. To test this hypothesis, we devised a pragmatic approach that expanded the pool of candidate molecules by leveraging the rapid peptide synthesis turnaround time and the ability to introduce modifications to the C- and N- terminus during synthesis.

To identify other sequence/modifications with oral activity, we designed a series of peptides composed of changes to the core RVTQ sequence and its evolutionary relatives. Additionally, we tested core sequences that included R > K changes in the first amino acid, V > I or L changes in the second position, T > S substitution in the third position, and Q > E in the fourth position resulting in 10 core peptide sequences. The core sequences were further modified to include the addition of miniPEG or acetyl group to the N-terminus, use of D-amino acids, and capping of the C-terminus with an amide group. We used Brown-Norway rats aged 60 to 136 days, which were gavaged twice per week with the various peptides. Each test was composed of three groups, a control and two peptide combinations, with a total of 34 peptides screened. We gavaged the various peptides with doses that ranged from 550 to 620 µg/kg, followed by blood sampling at two hrs. **Figure 4E** summarizes the peptides tested normalized to their controls testosterone levels and sorted accordingly to their statistical significance. The screening identified 11 core/peptide modifications that significantly increased testosterone levels, with the remaining showing no change or non-significant decreases. We confirmed that the animals still responded to the oral peptides by using RITQ, our first positive peptide, in the last test where significant increases in testosterone were observed (the latest peptide tested is denoted with *RITQ).

### 3.10 Dose-response studies and low dosing

To test the androgenic profile of the top 12 oral peptides at lower doses, we set up an experiment composed of four groups of Brown-Norway rats aged 82-131 days. The rats were gavaged twice per week with the various peptides covering the ~10, 220, or 420 µg/kg doses. The results from plasma samples collected two hours after the gavage showed that Ac-RITQdS-CONH_2_, KVSQ, RdVTQ, and Ac-RdITQ-CONH_2_ increased average testosterone levels, with some doses showing significance (**Figure 4F**, blue boxes). We identified several other peptides where testosterone levels decreased at the 10, 220 or 420 µg/kg doses (pink boxes). In addition, these results identified peptide candidates that increased testosterone levels around 420 µg/kg and at the low dose of 10 µg/kg, although not significantly. These data indicated that the most peptides were active at 550 to 620 µg/kg, and that some have a biphasic response at lower doses.

To gain further insights into the biphasic dose-response profile identified, we tested RITQ in rats gavaged each day with increasing concentrations from age 88 to 138 days. RITQ showed a biphasic profile for testosterone, characterized by a significant increase at the low dose of 10 µg/kg, a non-significant decrease at ~220 µg/kg, and a significant increase at ~520 µg/kg (**Figure 4G**), no corresponding significant changes in corticosterone levels were observed (**Figure 4H**).

### 3.11 Mix of two peptides at low-doses increase plasma testosterone levels after oral administration

The previous results showed that some of the peptides that increased circulating testosterone levels at ~420 µg/kg had a biphasic profile characterized by a repression trend of testosterone levels around the 220 µg/kg dose. The results also showed that the 10 µg/kg dose increased, although not significantly, average testosterone levels of 6 peptides. We hypothesized that a mix of two peptides may result in significant increases in testosterone levels, thereby avoiding potential negative effects observed in some of the peptides. To test this hypothesis, we treated Brown-Norway rats aged 91-134 days old with the possible 15 permutations generated from the mix of 6 candidate peptides dosed at 10 µg/kg each. **Figure 4I** summarizes the peptides tested normalized to their controls testosterone levels and presented sorted according to their statistical significance. The results show that 5 dual-peptide combinations showed significant increases in testosterone levels with the rest showing no changes in testosterone levels.

### 3.12 Modification to the core peptide increase the half-life in serum

To ascertain the stability of the leading candidate peptides in plasma, we incubated 4 µM of all peptides, except for KVSQ where we incubated with 12.5 µM, in 100 µl of plasma. The results showed that the half-life of the various peptides was as follows Ac-RITQdS-CONH_2_ > RdVTQ > Ac-RdITQ-CONH_2_ > RITQ > RVTQ > KVSQ (**Figure 4J**). As expected, peptide modification with end-capping or the introduction of D-amino acids had the longest half-life, while peptides that included K exhibited the fastest degradation.

We continued the pharmacokinetic characterization of Ac-RITQdS-CONH_2,_ RdVTQ, Ac-RdITQ-CONH_2_, RVTQ, and KVSQ *in vivo*. The results show that most of the peptides reach peak concentrations in the blood within 15 to 30 minutes ranging from 70 to 230 ng/ml (**Figure 4K**). The naked RVTQ and KVSQ cores were not detected in the rat plasma (data not shown).

In order to explain the observed oral absorption of RdVTQ, and analogs, following *in vivo* dosing as witnessed by the pharmacokinetic profile in **Figure 4K**, we utilized ADMET Predictor^®^ v10.3 to calculate critical physicochemical properties of the peptide. The oil/water partition coefficient and ionization constants for acidic and basic functional groups, often expressed as logP and pKa, resp., correlate to oral biopharmaceutics due to their relationship to drug solubility and permeability (59). Detailed model output of predicted macrostates and dissociation constants for respective ionized forms of RdVTQ are shown (**Table 1**). Specifically, the basic pKa of 7.8 illustrates the biorelevant pKa value for RdVTQ which differentiates this peptide from analogs. The two distinct basic pKa’s for RdVTQ govern net positive ionization in gastrointestinal tract (gastric pH of ~1.2 to intestinal pH of 6.8-7.8, i.e., from proximal duodenum to ascending colon), as well as lower logD_pH7.4_ (distribution coefficient) following oral dosing. RdVTQ maintains a net 2+ to 1+ charge, from gastric to small intestinal pH, sensitive to the free carboxylic acid terminal (C-terminal). C-terminal is protonated at low stomach pH resulting in RdVTQ^2+^, while in small intestine it gets deprotonated resulting in RdVTQ^1+^. Maintenance of a net charge supports keeping RdVTQ in a dissolved state after oral dosing, while analogs Ac-RITQdS-CONH_2_ and Ac-RdITQ-CONH_2_ will maintain a net positive state. Furthermore, the calculated logP of -3.3 also suggests at least 5-20 times higher hydrophilicity as seen in predicted 1.6-to-3.8 times higher intrinsic solubility for RdVTQ vs. analogs. Remarkably, the solubility at pH7.4 for the N- and C-terminal modified analogs is 1.6-2.6 times higher than that of RdVTQ, likely attributes to the lack of acidic pKa’s. Gastric emptying may facilitate intestinal permeability as suggested by the predicted effective jejunal, apparent MDCK, and fraction of *in vivo* oral dose (10 mg) absorbed. Following absorption, all peptides are predicted to have a large, >85%, fraction unbound in plasma (**Table 1**).

**Table 1:** Calculated physicochemical and biopharmaceutical properties of peptides tested *in vivo* rat pharmacokinetic studies.

	Identifier	Formula Weight	Acidic pK_a_	Basic pK_a_	log P	logD pH_7.4_	P_eff_**	Intrinsic solubility (aq), mg/mL	Solubility pH_7.4_, mg/mL	rat f_up_%	P_app_ MDCK^***^	rat %Fa (10mg) ^#^
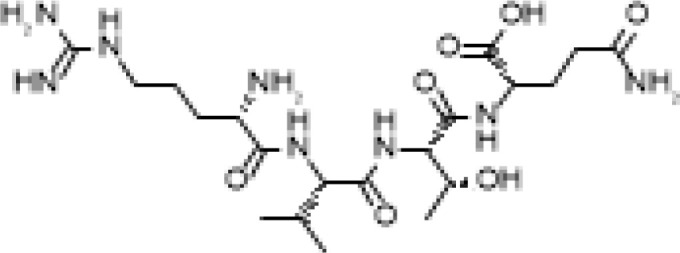	RdVTQ	503	3.81	12.61; 7.80	-3.3	-3.6	0.12	45	156	84.5	7.1	13.0
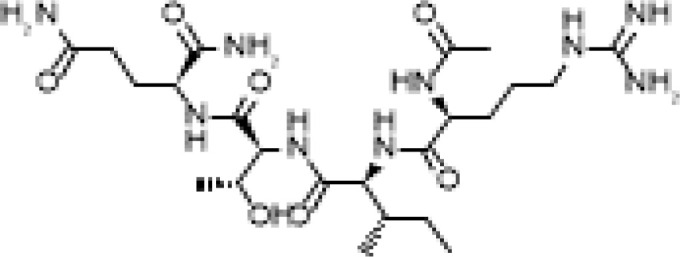	Ac-RdITQ-CONH2	558	None	12.4	-2.0	-2.2	0.07	12	264	85.7	12.8	8.0
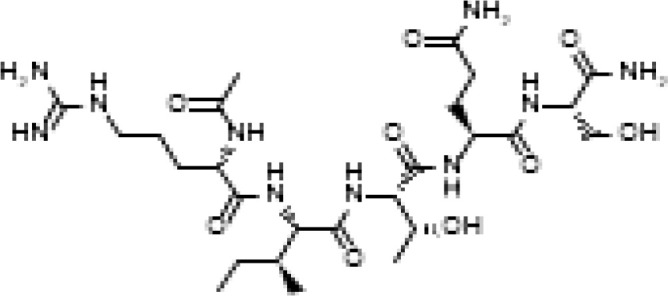	Ac-RITQdS-CONH2	645	None	12.4	-2.6	-2.6	0.05	29	408	87.4	10.7	6.3

**Effective jejunal permeability (cm/s × 10^4^).

***Apparent MDCK Transwell^®^ permeability (cm/s x 10^7^).

^#^Fraction absorbed in selected species at specified (10mg) dosage level.

### 3.13 RdVTQ increases consistently testosterone levels

**Figure 5:**
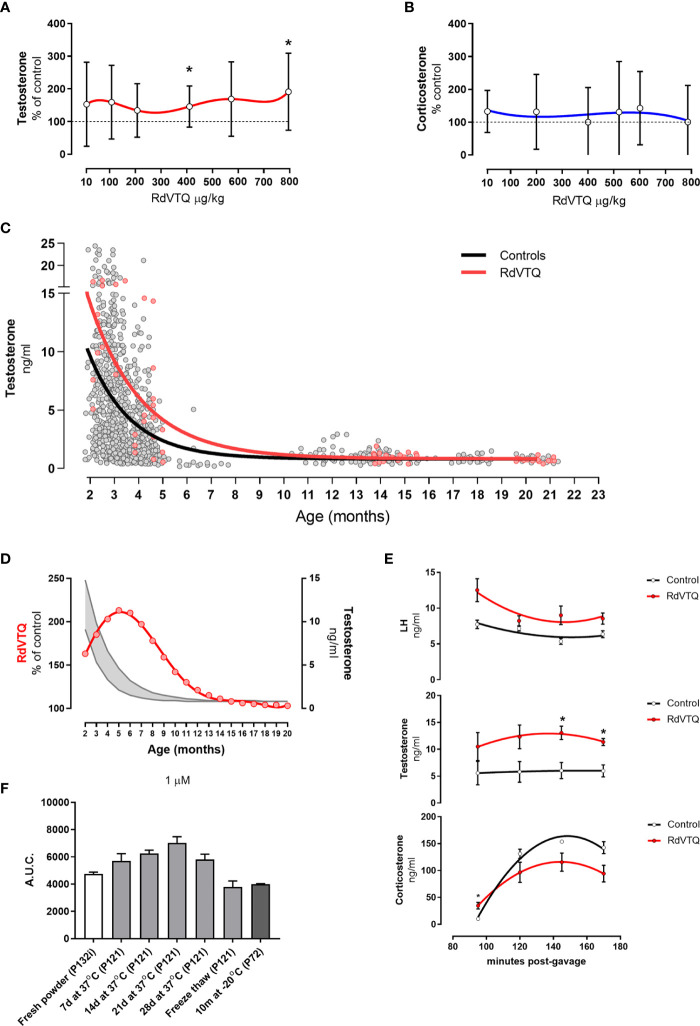
**(A-B)** Dose response shows biphasic response in some peptides. **(A)** Testosterone and **(B)** corticosterone levels of Brown-Norway rats 2 hrs after gavage with the various concentrations of RdVTQ. Age of Brown-Norway rats during experiment was 88-138 days-old; N = 8; results shown as mean ± SD; *p<0.05. **(C)** Circulating testosterone levels in Brown-Norway rats under various conditions and ages. Testosterone levels in rats gavaged with water (control; black line) or 550 μg RdVTQ/kg (red line). Plasma samples were collected 2hrs post-gavage. Plasma samples were collected 90 min post-intraperitoneal injection. The lines represent the equation that best-fits the data collected. Each dot represents the average of two measurements per sample. Control (black line) includes 897 dots and RdVTQ (red line) includes 62 dots. **(D)** Effect of RdVTQ on circulating testosterone levels throughout the lifetime. Grey lines represent the average testosterone levels of rats gavaged with control (water; grey lower line) or RdVTQ (550 μg/kg; grey upper line). Red line represents the projected RdVTQ-driven percent testosterone level increase throughout the rat lifetime. These data were calculated from the best-fit analysis. **(E)** Effect of RdVTQ on circulating LH, testosterone, and corticosterone levels in selected timepoints after gavage. Sprague-Dawley rats were gavaged with 550 μg RdVTQ/kg (0 minutes) followed by jugular plasma collection at the denoted time points. N = 4; results shown as mean ± SEM; *p<0.05. **(F)** Stability of RdVTQ under various conditions. RdVTQ was maintained at 37°C for the denoted timepoints, submitted to three cycles of freeze thaw, or stored for 10 months at -20°C. Stability of each condition was ascertained by mass-spec quantification. Area under the curve (AUC) was plotted for each condition.

The data showed that RdVTQ, a close derivative of the naked RVTQ core, consistently and significantly increased plasma testosterone levels in the first screen (**Figure 4E**), in the dose-response experiment (**Figure 4F**), and in the dual-peptide experiment (**Figure 4I**). RdVTQ also showed a stable profile during the serum and *in vivo* pharmacokinetic characterization. Thus, we chose to further characterize RdVTQ. Dose-response studies showed that RdVTQ significantly increased testosterone levels starting at >400 µg/kg (**Figure 5A**). We observed an increased trend in testosterone levels, although not significant, in the lower 10 µg/kg. RdVTQ lacked the repressive profile of other peptides near the 200 µg/kg dose (**Figure 5A**). No changes in corticosterone were observed during the dose-response experiment (**Figure 5B**).

We characterized the ability of RdVTQ to increase testosterone levels during most of the life span of the Brown-Norway rats. **Figure 5C** shows the pool testosterone levels of control rats (grey), and rats that were orally treated with RdVTQ and plasma samples collected two hours after (red). The results show that RdVTQ increases plasma testosterone levels in the young to mature adult and that Brown-Norway rats show a limited response after 10 months of age. The performance of RdVTQ is maximal in 5-month-old rats and gradually decreases there after (**Figure 5D**).

We characterized the kinetics of testosterone, corticosterone, and LH during a window of 90 to 170 minutes after a single oral gavage of ~550 µg/kg of RdVTQ (**Figure 5E**). Samples were collected at 90, 120, 150, and 170 minutes. The data showed that testosterone levels are the highest between 120 to 150 minutes post RdVTQ gavage. LH levels in RdVTQ treated rats showed an inverse correlation with peak testosterone levels and an upward trend towards the 170 minutes. Corticosterone levels were the lowest during the first percutaneous jugular sample and increased proportionally thereafter with no significant changes.

RdVTQ stability was tested by measuring the area under the curve of fresh peptide, peptide solubilized in distilled water and incubated at 37°C for 7 - 28 days, peptide frozen and thawed for 6 cycles, and peptide stored at -20°C for 10 months. The results showed that RdVTQ is highly stable. We observed a minor decrease in the area under the curve in peptide that was freeze/thawed and stored at -20°C for 20 months (**Figure 5F**).

## 4 Discussion

We previously reported that a 27 amino acids peptide, originally known as TV167, composed of a TAT-cell penetrating sequence and a fragment of VDAC1 increased testosterone formation *in vitro*, *ex vivo*, and when infused intratesticularly for 24 hr (56). Based on these data, we hypothesized that TV167 was disrupting protein-protein interactions known to regulate the influx of cholesterol into the mitochondria, which ultimately increased testosterone production (49). The data also suggested that the 13 amino acids sequence located between the cytosolic 10th and 11th transmembrane loops of VDAC1 was an important region involved in controlling the influx of cholesterol into the mitochondria. While our previous findings were significant in identifying a new pro-androgenic molecule, they were limited by the unfeasible route of delivery and the high potential for hypothalamic-pituitary-gonadal (HPG) negative feedback regulation in deliveries extending more than 24 hrs.

Here, we set out to further characterize TV167, now TV159-172, with the overarching goal of creating a drug derivative with a more favorable pharmacokinetic profile and potential for future clinical development. Based on our goal, this body of work had the following milestones (i) to further characterize the HPG interactions of TV159-172 using a subcutaneous delivery model, (ii) to identify the active core sequence using deletion experiments, and (iii) to generate small molecule derivatives with more favorable pharmacokinetics that can be administered orally. We favored a highly pragmatic approach that leveraged the rapid generation of small peptides coupled with an efficient *in vivo* screening method that used circulating testosterone levels as a biomarker. While the generation of the peptides was not based on traditional computation-based methods, our approach bypassed the resource-intensive challenges associated with traditional *de novo* small drug development.

We started by improving the route of administration of TV159-172 from a 24hr intratesticular delivery used in our previous work to a one-week subcutaneous delivery in the Sprague-Dawley rat and performed dose-response studies. The testosterone biomarker data identified 30 to 83 ng/kg/day as a dose-range where TV159-172 was active, which was close to the ~17 ng/kg/day intratesticular dose used in our previous study (49). While in the first gross experiment we noted an increase in corticosterone levels, the expanded experiment showed no changes or a decreased trend. This may be due to the sampling conditions that not favorable to measuring corticosterone.

We characterized the effect that a 42-day subcutaneous infusion of TV159-172 had in the HPG axis. The results showed that testosterone and LH levels mirrored each other with significant changes only observed at infusion day 35 and not at 15. This observation extends our previous findings where a 24 hrs infusion increased testosterone while decreasing LH levels (49). As expected, the data suggested that the prolong infusion interacted with the HPG resulting in fluctuating steroid levels. In fact, the repression of testosterone levels was the predominant challenge we faced in Sprague-Dawley rats throughout this body of work. Despite the fluctuating testosterone levels, the significant increase in steroid levels, independent of the collection time, corroborated that TV159-172 was active. These data also showed that the physiological HPG feedback regulation is a “smart” safety mechanism that protected against prolonged supraphysiologic testosterone levels. TRT is widely misused and the development of a new therapeutic that inherently prevents abuse is a significant achievement (60–62).

We continued the characterization of the HPG axis role on TV159-172 action. The results showed that TV159-172 did not stimulate *in vivo* steroid production past the maximum obtained when rats were suprastimulated with hCG or ACTH. The results also showed that TV159-172 did not stimulate, by itself, steroid levels in hypophysectomized animals. This lack of effect may be due to Leydig cell deterioration from the prolonged LH suppression (63–66), or from a pan-hormone suppression secondary to the loss of anterior and posterior pituitary, or because partial LH signaling was needed for the peptide to function. In our previous work, we observed that TV159-172 increased testosterone levels in a GnRH antagonist-induced chemically castration model and interpreted the results to suggest that TV159-172 directly increased androgen levels (49). Here, the hypophysectomized data suggest that the low LH levels, which were not completely removed by the chemical castration (67), were sufficient for TV159-172 action. Taken together these data suggest that TV159-172 action requires the Leydig cell to have functional infrastructure, e.g. endoplasmic reticulum, which depends on the trophic effect of LH, and that TV159-172 likely acts as an LH-signaling amplifier.

We explored whether TV159-172 would be permissive to deletions to the VDAC1-portion of the peptide. We generated TV160-172, TV160-171, and TV160-170 bearing small deletions and infused them for one-week. The biomarker data showed that TV160-171 and TV160-170 resulted in meaningful increases in testosterone at selected peptide dose levels indicating that further deletions to the VDAC1 sequence could be feasible.

In a follow-up experiment, we explored whether the shorter TV160-171 and TV160-170 peptides could increase testosterone levels for a sustained period. The data showed that both peptides increased circulating testosterone levels until infusion day 73, with most rats having 2-3-fold increases. Interestingly, some rats reached upwards of 10-fold increases suggesting that the peptides can potently stimulate testosterone, but independently of the levels achieved, the effect of the TV-peptides was diminished by infusion day 88. These data corroborated that the HPG axis acted as a “safety guider” that eventually modulated testosterone levels within a physiological range. The different doses at which the peptides were active indicated that the deletions unpredictably affected peptide dosing and that dose-response studies were needed to continue the deletion studies. Moreover, the suppression of the peptide effects suggested that continuous subcutaneous infusion was an unlikely therapeutic option. Despite this, the subcutaneous model gave insights into the HPG response and became an efficient method of identifying bioactive peptides.

The previous experiments indicated that deletions in the TV159-172 sequence were feasible and that the subcutaneous model can be used to identify the bioactive core. We used Brown-Norway rats to find the core using dose-response experiments and progressive deletions of the VDAC1-part of the fusion peptide in the subcutaneous model. The data showed that TV163-167 and TV162-166, which retained a 5 amino acid VDAC1-sequence, were the shortest TV-bearing peptides that were steroidogenic active. We further tested whether N163-166 (Naked RVTQ), which corresponds to the overlapping sequence of TV163-167 and TV162-166, increased testosterone levels. These results indicated that RVTQ was the active core, that the TAT-cell penetrating peptide was not needed for cell delivery, and that this short sequence did not affect corticosterone levels. This significant finding allowed us to use RVTQ as template to design small molecule compounds with the potential to be orally administered.

The dose-response graphs that included all the TV and the N163-166 peptide showed that, independent of sequence, the peptides were androgenic at doses >100 ng/kg/day. Moreover, we pooled testosterone levels data independent of the dose to identify the overall activity of the TV deletions. The pooled analysis of control rats composed of 60 samples yielded a rat with 2.6 times the pooled control average as the control rat with the highest testosterone levels. In contrast, all peptides tested had several rats with testosterone levels beyond the highest control rat, with some rats reaching upwards of 5-fold increase above the pooled control average. While the pooled results were not meant to be used for statistical analysis, they showed segregation of rats (i.e. TV160-169 and TV162-169) into low and high levels responders, suggesting that some rats reach testosterone levels that triggered modulation by HPG axis within the 7-day duration of the study. In agreement with the Sprague-Dawley rat data, a pattern of testosterone inhibition in Brown-Norway rats was observed in TV159-172 at 30 ng/kg/day, where most rats had plasma testosterone levels 50% below those of the control average.

Measurement of plasma corticosterone levels in the same Brown-Norway rats treated with the various peptides indicated peptide-specific activity. The longer TV160-171, TV160-170, and TV162-169 peptides promoted significant increases in plasma corticosterone levels, while the shortest TV163-167 and TV162-166 showed no significant changes. Some corticosterone levels, like those seen in TV160-170, were higher than those seen in testosterone measurements and were upwards of 15 times those of the control average. In agreement with our previous data, these results suggested that some rats showed very high corticosterone levels because, unlike testosterone, corticosterone is not as tightly regulated (68). These data must be interpreted with caution since the percutaneous collection of the blood samples was not the optimal method to evaluate adrenal effects. This is because the collection process affects the stress levels and, consequentially, corticosterone release in these rats. Despite the suboptimal collection, the effects observed in TV160-171 and TV160-170 show a dose-response effect. In summary, the data indicated that the length of the TV159-172-derived sequences had differential effects on testicular vs adrenal-made steroids, with longer sequences favoring corticosterone production while shorter sequences targeting Leydig cell testosterone biosynthesis.

Using the subcutaneous model, we confirmed that the RVTQ (N163-166) core was active in Brown-Norway rats and found a two-fold increase in plasma testosterone levels in ~54-day-old Brown-Norway rats. We started the development of small molecule compounds by ascertaining if the core was permissive to modifications. We used an evolutionary approach to study whether substitutions with related amino acids to the RVTQ core retained steroidogenic activity. Sequence analysis of all VDAC1 entries found in the UniProt portal identified RITQ, KITQ, and RVSQ as core-variations found in nature. These substitutions found in nature were of structurally related amino acids. The data showed that infusion of RITQ and KITQ significantly increased testosterone levels, indicating that changes to the protein core were feasible. These data also suggested that the various synthetic peptides interacted with the HPG axis since, for example, subcutaneous delivery of RITQ showed a non-significant increase that became significant during our second round of measurements.

We then synthetically modified the RVTQ core with various changes. Because we do not yet fully understand the mechanism of action of the peptide, we initially hypothesized that a perfect interaction of the RVTQ core with VDAC1 might elicit a full response, and thus, be more prone to negative feedback. This would explain some of inconsistent responses that we sometime observed. Indeed, the peptide-initiated mechanism of action is so potent that naked peptides at very low doses can also elicit the response. Examples of these naked peptides that lack modifications are RITQ, KVSQ, RISQ (**Figure 4E**) and RITQ + KVSQ (**Figure 4I**) with increased response at lower drug levels. The fact that the above naked peptides can trigger an oral response is a strong indicator of the presence of on/off switch activated by these molecules. Therefore, the modifications were undertaken to decrease the potency of the core by introducing small changes in structurally related amino acids guided by evolution (otherwise the combinations would be endless). In addition, the modification introduced to the RVTQ core increased the half-life of peptides since the rapid peptide degradation is the main challenge for the use of peptides as oral therapeutics (69, 70).

To screen for oral candidates, we developed an animal model where Brown-Norway rats were gavaged with the various RVTQ core modifications followed by percutaneous jugular blood sampling arbitrarily set at three hours post-gavage. We used the native RVTQ sequence as a control and administered the modified core derivatives using arbitrary doses. The data showed that addition of a D-serine and capping of the carboxy terminus with NH_2_ resulted in a significant increase in testosterone levels. This finding was confirmed in two follow up experiments that characterized the best sampling time point, which was stablished at 2 hrs post-gavage with ~580 µg/kg of RVTQdS-CONH_2_. These findings were substantial since an increase in steroid levels during a 2 to 3 hrs window is likely to escape HPG repression and allow for multiple dosing that mimics the physiological circadian peaks in testosterone production.

We expanded the search for additional molecules using Brown-Norway rats to test various peptides and established blood collections at 2 hrs post-gavage with peptide dosing that ranged from an average of 530 to 620 µg/kg. This oral screening included molecules with structurally-related amino acid modifications to the RVTQ core where the R was substituted with K, V was substituted with I or L, T was substituted with S, and Q was substituted with E. These amino acid modifications generated 10 core sequences that, when combined with D-amino acids, capping of the peptide ends, and use of mini-PEG, generated 34 candidate small molecules. The screening identified 11 small molecules that significantly increased testosterone levels. To confirm that the effects were still present at the end of our screening, we treated the rats at the end of the experiment with RITQ, the first molecule identified, and observed significant increases in testosterone. Moreover, this screening started when the rats were ~60 days-old; however, the data showed that ~80-90-day-old rats were a better starting point, since younger rats exhibit higher testosterone levels that may mask the peptide effect. We were surprised by the range of amino acid changes and protein modifications that increased testosterone levels, which included the unmodified cores RITQ, KVSQ, RISQ, KITQ, RVSQ, and SRVTQ (underlined letters denote amino acid changes from the original RVTQ core). In general, modifications to V were the most permissive except for L where the additional carbon may have resulted in steric interference. The data also suggested that Q was essential since any substitution abolished peptide activity in agreement with our initial evolutionary-guided analysis where Q was conserved among all the sequences processed. We also identified several other peptides that showed a non-significant trend to increase testosterone levels and hypothesized that some of these peptides might be active if the dosing was adjusted or administered at an earlier time point. Moreover, we identified several other peptides with a non-significant trend to decrease testosterone levels that might indicate a dose-dependent repression state.

We then wanted to identify the peptides that robustly increased testosterone levels across the doses. We improved the testing conditions by using rats older than 80 days, collecting blood samples 2 hrs after gavage, and dosing at ~10, 220, and 420 µg · peptide/day. We tested the 11 molecules that significantly increased testosterone, including Ac-RITQdS-CONH_2_, the first peptide excluded from the inclusion criteria but that showed a trend to increase testosterone levels. This screening identified Ac-RITQdS-CONH_2_, KVSQ, RdVTQ, and Ac-RdITQ-CONH_2_ as those peptides that consistently increased testosterone levels across the doses tested. We identified several other peptides that showed a decrease in steroid levels at ~220 µg/kg, which included RITQ, in agreement with follow-up dose-response studies that measured androgen and corticosterone levels. In summary, this dose-response screening identified 4 peptides with robust profiles and, in addition, the data showed that dosing at ~10 µg/kg increased, although not significantly, the average testosterone levels of Ac-RITQdS-CONH_2_, RdVTQ, Ac-RdITQ-CONH_2_, RITQ, KVSQ, and Ac-KdITQ-CONH_2_.

We then sought to improve the efficiency of selected peptides to decrease dosage used while increasing safety margins. We tested whether a combination of two of the 6 peptides, which due to their modifications may have different pharmacokinetic profiles, could act together to exhibit significant effects. We selected Ac-RITQdS-CONH_2_, KVSQ, RdVTQ, Ac-RdITQ-CONH_2_, RITQ, and the original RVTQ core tetrapeptide. Permutation of these 6 small molecules, composed of 3 native and 3 modified cores, resulted in 15 possible 2-peptide combinations that were tested. We set the testing conditions as follows: Brown-Norway rats age >90 days old, blood collection 2 hrs after gavage, and used 10 µg/kg each of the 2 peptides tested. The results identified 5 combinations that significantly increased testosterone levels. This finding represented a ~55-fold improvement from our first and second screenings set at ~550 µg/kg. The very low doses used in the oral 2-peptide mixes are likely to avoid side effects, be cleared rapidly, facilitate repetitive dosing, and decrease manufacturing costs.

The pharmacokinetic profile of 6 leading peptides in plasma and *in vivo* indicated that the modification to the core significantly increased the peptide half-life. The data showed that most of the detectable peptides reached peak concentrations 15 - 30 min post-gavage with Ac-RITQdS-CONH_2_ being the fastest absorbed in ~15 min. These data, which showed rapid clearance of the peptides, agrees with the finding that testosterone levels reached peak induction levels at 2 hrs. Moreover, we found evidence showing that the peptide half-life and that the peptide levels in serum do not correlate with the androgen response supporting an on-off mechanism. In the *in vivo* PK experiments, to reach detectable levels, we used a single 10 mg dose in 1 ml of vehicle, which was ~20 times the therapeutic mono-dose. Despite the larger dose, we could not detect RITQ, RVTQ, or KVSQ for which the input peptide was 4-times greater than all other peptides tested. The rapid degradation of KVSQ indicated that the lysine significantly increased peptide degradation in our *in vitro* assay and presumably *in vivo*. Moreover, the rapid degradation of KVSQ in our *in vitro* and *in vivo* assays is surprising since this tetrapeptide seems to activate steroidogenesis consistently. The bioactivity and rapid degradation of KVSQ suggest that only few molecules are needed to activate the molecular target and elicit effects. The observed oral absorption (**Figure 4K**) of RdVTQ following *in vivo* dosing correlates well to drug solubility and permeability parameters identified based on the predicted macrostates and dissociation constants for the respective ionized forms of RdVTQ (**Table 1**), keeping the peptide in a dissolved state after oral dosing.

We then selected RdVTQ for further characterization because it consistently increased testosterone levels, had one of the smallest molecular weights, and was closest to the RVTQ core. In agreement with our previous screening data, the RdVTQ dose-repose experiments showed significant androgen activity that did not impact corticosterone levels. To characterize the therapeutic age window of RdVTQ, we pooled data from all our control animals either treated with orally with water or surgically implanted with pumps delivering water. The data revealed that RdVTQ had the greatest performance in the adult rat and that the peptide performance rapidly decreased after 7 months of age. These age ranges roughly correspond to the period in which testosterone starts to decline in humans. However, it is difficult to compare rats with humans in terms of testosterone levels. Although a 6-month-old rat corresponds to an 18-years-old human and a 18 months old rat to a 45 years old human (http://www.ratbehavior.org/RatYears.htm), in humans, testosterone begins declining after 30 years of age (4–6) whereas testosterone levels in rats begin declining after 3 months of age in rats (71). Moreover, the experiments also showed that corticosterone levels were not significantly affected by RdVTQ.

It is important to note that this body of work was conducted in two species of healthy rats where the peptides consistently achieved a boost in testosterone levels. While maximal peptide activity in healthy rats was achieved up to 7-8 months, the data suggest that the peptides act as LH-signaling amplifiers with the potential to improve the magnitude and duration of the effect in human disease states such as hypogonadism. Based on the initial PK/PD profile, RdVTQ has the potential of being administered early in the morning and in the afternoon to achieve a physiological-like boost in testosterone levels while having an expected favorable safety profile.

In summary, we developed a first-in-class family of small molecules that act within the HPG axis to increase testosterone levels. The data indicate that the therapeutic use of the peptide might recover Leydig cell function in the young and slow down the effects of aging in Leydig cell testosterone formation while maintaining a high safety profile.

## Data availability statement

The original contributions presented in the study are included in the article/**Supplementary Material**. Further inquiries can be directed to the corresponding author.

## Ethics statement

Animals were handled according to protocols approved by the McGill University Animal Care and Use Committee.

## Author contributions

DM-A contributed to conceptualization, methodology, formal analysis, investigation, data curation, writing the original draft, review, and editing. JN and HG contributed to methodology and data analysis, and VP contributed to conceptualization, formal analysis, resources, review and editing, supervision, project administration, and funding acquisition. All authors contributed to the article and approved the submitted version.

## Supplementary material

Supplementary Figure S1 Dose scan of TV159-172 infused subcutaneously. Sprague-Dawley rats were implanted with osmotic pumps delivering various concentrations of TV159-172 for 1 week. Levels of intratesticular testosterone **(A)**, serum testosterone **(B)**, dihydrotestosterone **(C)**, luteinizing hormone **(D)**, estradiol **(E)**, corticosterone **(F)**, and aldosterone **(G)** were measured in day 7 as described under material and methods. Data shown represent means ± standard deviation (N=5).

Supplementary Figure S2 Modifications to the RVTQ core increase plasma testosterone levels after oral administration. A) Testosterone levels in Brown-Norway rats 3 hrs after gavage with RVTQ and indicated RVTQ derivatives used at the indicated concentrations. Age of Brown-Norway rats during experiment was 60-123 days-old; N = 7; results shown as mean ± SD; *p<0.05; T denotes the order with which the peptides were tested.
