# Supplementary material for: Oral administration of VDAC1-derived small molecule peptides increases circulating testosterone levels in male rats
Source: Front Endocrinol (Lausanne). 2023 Jan 4;13:1003017. doi: 10.3389/fendo.2022.1003017 (PMC9846164; doi:10.3389/fendo.2022.1003017)
Supplement: Supplementary Figure S1 — Dose scan of TV159-172 infused subcutaneously. Sprague-Dawley rats were implanted with osmotic pumps delivering various concentrations of TV159-172 for 1 week. Levels of intratesticular testosterone (A), serum testosterone (B), dihydrotestosterone (C), luteinizing hormone (D), estradiol (E), corticosterone (F), and aldosterone (G) were measured in day 7 as described under material and methods. Data shown represent means ± standard deviation (N=5). [file Presentation_1.pdf]

Supplemental Data

Oral administration of VDAC1-derived small molecule peptides increases circulating testosterone levels in rats

Daniel B. Martinez–Arguelles[1], Jennifer W. Nedow[1], Hovhannes J. Gukasyan[2] and Vassilios Papadopoulos[1,2*]

[1]Research Institute of the McGill University Health Centre and Department of Medicine, McGill University, 1001 Decarie Blvd, Bloc E, Montreal, Quebec, H4A 3J1, Canada.
[2]Department of Pharmacology and Pharmaceutical Sciences, School of Pharmacy, University of Southern California, 1985 Zonal Avenue, Los Angeles, California 90089, USA.

Supplemental Figures: 2

Figure S1. Dose scan of TV159-172 infused subcutaneously. Sprague-Dawley rats were implanted with osmotic pumps delivering various concentrations of TV159-172 for 1 week. Levels of intratesticular testosterone (A), serum testosterone (B), dihydrotestosterone (C), luteinizing hormone (D), estradiol (E), corticosterone (F), and aldosterone (G) were measured in day 7 as described under material and methods. Data shown represent means ± standard deviation (N=5).

Figure S2. **Modifications to the RVTQ core increase plasma testosterone levels after oral administration.** A) Testosterone levels in Brown-Norway rats 3 hrs after gavage with RVTQ and indicated RVTQ derivatives used at the indicated concentrations. Age of Brown-Norway rats during experiment was 60-123 days-old; N = 7; results shown as mean ± SD; *$p<0.05$; T denotes the order with which the peptides were tested.

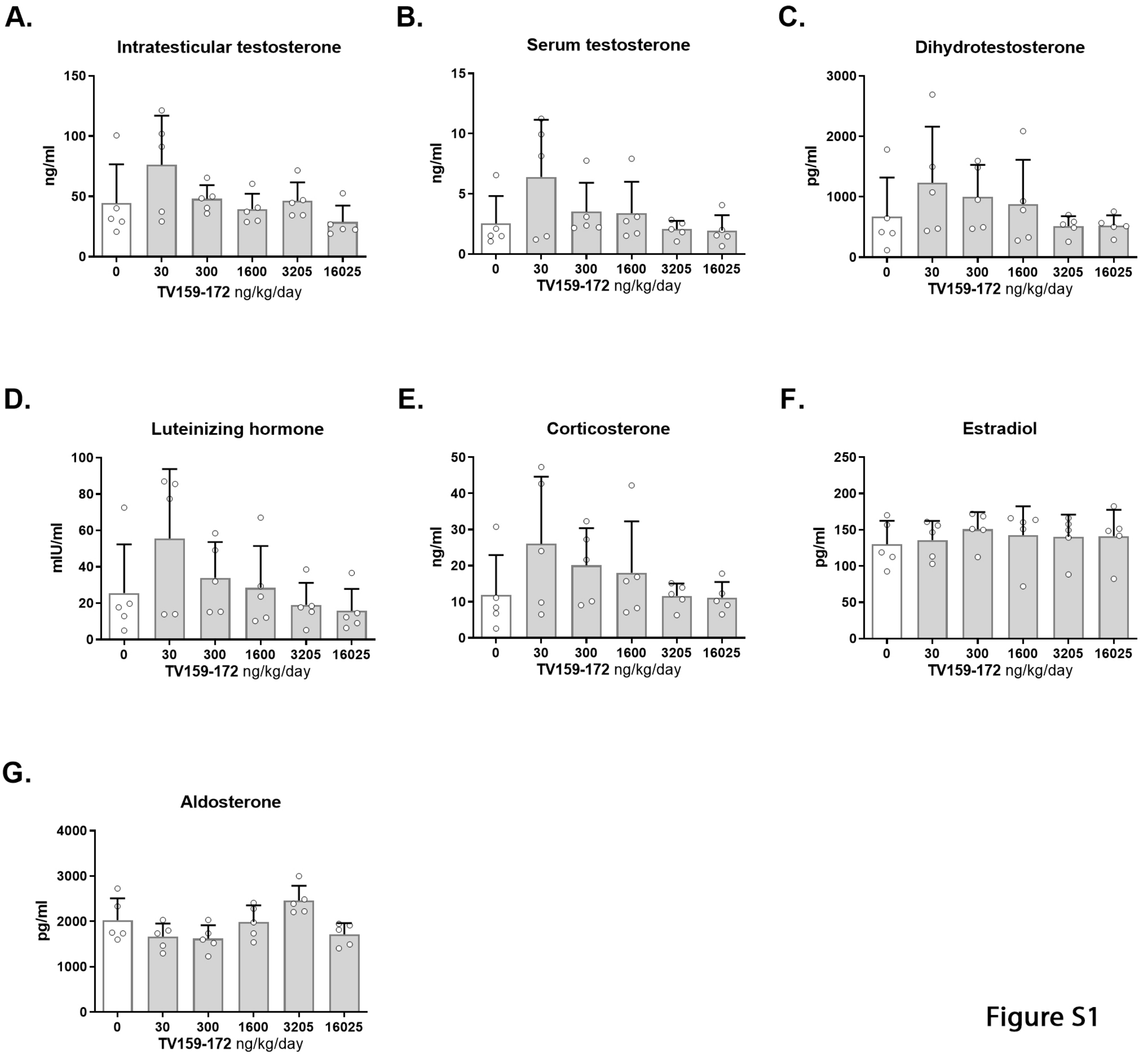
A.
Intratesticular testosterone
ng/ml
B.
Serum testosterone
ng/ml
C.
Dihydrotestosterone
pg/ml
D.
Luteinizing hormone
mIU/ml
E.
Corticosterone
ng/ml
F.
Estradiol
pg/ml
G.
Aldosterone
pg/ml
0 30 300 1600 3205 16025
TV159-172 ng/kg/day

Figure S1

Figure S2

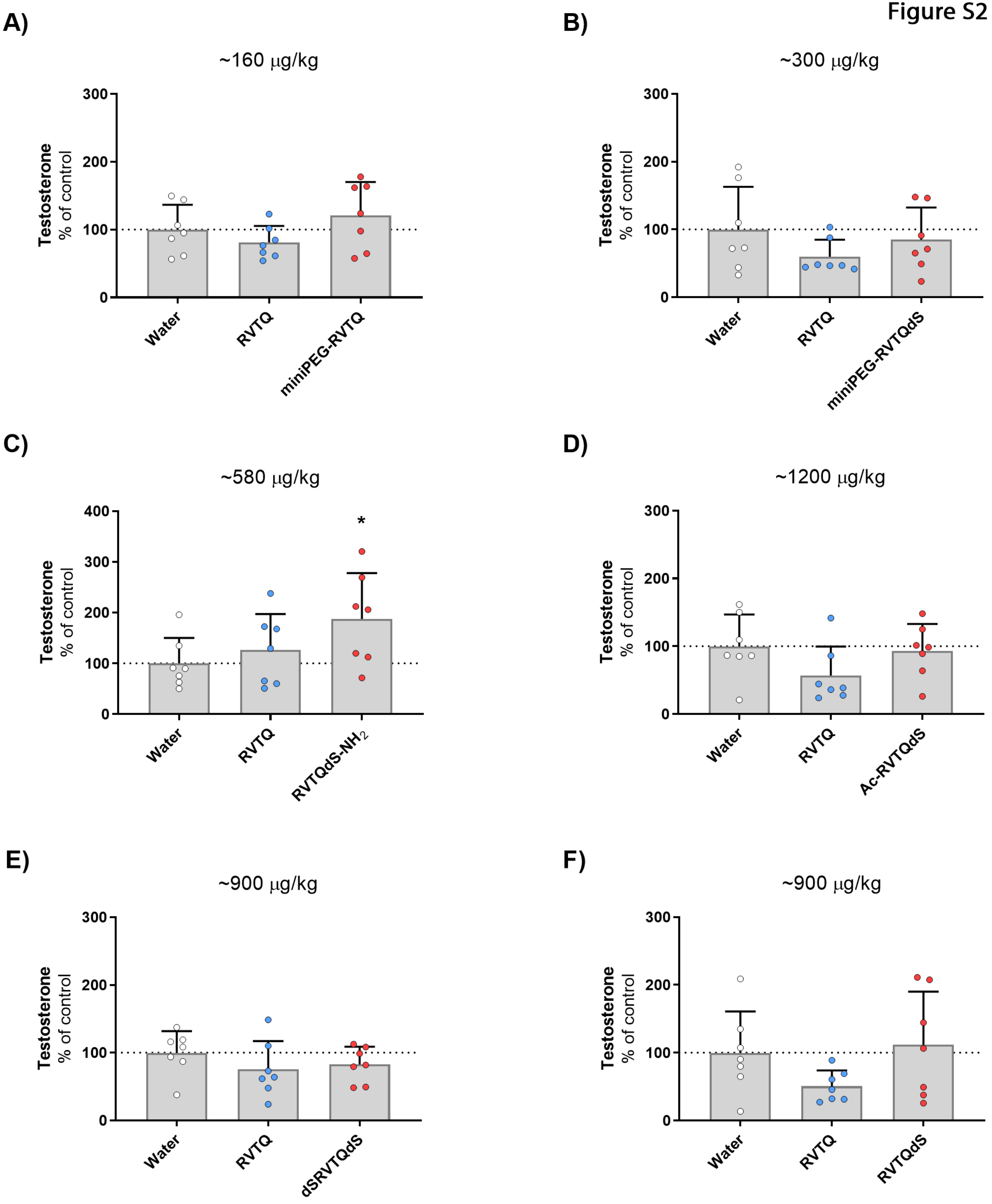
A)
~160 μg/kg
Testosterone
% of control
300
200
100
0
Water
RVTQ
miniPEG-RVTQ
B)
~300 μg/kg
Testosterone
% of control
300
200
100
0
Water
RVTQ
miniPEG-RVTQdS
C)
~580 μg/kg
*
Testosterone
% of control
400
300
200
100
0
Water
RVTQ
RVTQdS-NH2
D)
~1200 μg/kg
Testosterone
% of control
300
200
100
0
Water
RVTQ
Ac-RVTQdS
E)
~900 μg/kg
Testosterone
% of control
300
200
100
0
Water
RVTQ
dSRVTQdS
F)
~900 μg/kg
Testosterone
% of control
300
200
100
0
Water
RVTQ
RVTQdS
